# Using Music to Develop a Multisensory Communicative Environment for People with Late-Stage Dementia

**DOI:** 10.1093/geront/gnz169

**Published:** 2019-12-08

**Authors:** Amy Clare, Paul M Camic, Sebastian J Crutch, Julian West, Emma Harding, Emilie Brotherhood

**Affiliations:** 1 Salomons Institute for Applied Psychology, Canterbury Christ Church University, Kent, UK; 2 Queen Square Institute of Neurology, Dementia Research Centre, University College London, UK; 3 Royal Academy of Music, London, UK

**Keywords:** Dementia, Communication, Live music, Multisensory, Residential care

## Abstract

**Background and Objectives:**

Research has indicated the benefit of music interventions on biological, psychological, and cognitive aspects of dementias, yet there is limited research focusing on music’s role in communication. This study developed a conceptual understanding of how people with late-stage dementia may express themselves nonverbally and interact with others during a live music group over time.

**Research Design and Methods:**

Eight people with advanced dementias in residential care (aged 82–97 years), four care staff, and three musicians participated in 8-hr-long weekly live Music for Life sessions and listened to 1-hr-long recorded music session. Visual grounded theory was used to analyze video data collected nonintrusively via the Fly 360-degree camera.

**Results:**

The live music group facilitated a multisensory communicative environment allowing for verbal and nonverbal communicative actions, social interactional components and agency to develop over time. These aspects were influenced by three factors: time, one-to-one interaction within a group setting and the characteristics of the music.

**Discussion and Implications:**

Nonverbal communication in later-stage dementia may be overlooked or underestimated by busy care staff and families. Using music as an interactive way to communicate can help develop mirroring and turn-taking which has been shown to improve quality of life for people with communication impairment, increase their nonverbal communication and allow for a connection to be built between people. Although further research is recommended, individuals responsible for residential care should feel confident that the development of ongoing music groups for this population is warranted as part of ongoing care.

The field of dementia care has been greatly influenced by Kitwood’s (1997) theory of “personhood,” which proposed that the needs of people with dementia include comfort, attachment, inclusion, occupation, and identity. Although Kitwood’s ideas have been hugely influential over time there has been a move towards more relational theories of dementia. Nolan, Davies, Brown, Keady, and Nolan (2004) claim that person-centered care does not adequately capture the reciprocity and interdependency that underlies caring relationships and that it needs to be expanded to a relationship-centered care approach underpinned by a psychosocial theory of relating they identified as the “senses framework.” Within this framework, Nolan and colleagues (2004) suggest that all parties involved in caring need to experience relationships that promote a sense of security, belonging, continuity, purpose, achievement, and significance. According to Henwood and Ellis (2015), dementia treatment and care has historically tended to focus on physical rather than social aspects. de Oliveira and colleagues (2015) found that nonpharmacological interventions (e.g., music interventions) have significant impact on biological, psychological, and cognitive symptoms of dementia and shifting focus to more relational aspects, therefore they should be considered as a primary intervention.

## Communication

According to Ridder and Gummesen (2015), people with dementia, particularly those who may have difficulty with verbal communication, may be at increased risk of social isolation and perceived as noncommunicative or even not existing. Social bonding is built upon communication and it is not just a linguistic message that is exchanged within an interaction but also the equally important paralinguistic and nonverbal messages (Ridder, 2003). Gesture, prosody and exaggerated expression offer the cues within an interaction that help turn-taking take place for people with severe communication difficulties (Holck, 2004) and therefore are the essential building blocks to creating a relationship.

A recognition of the impact of social isolation on people with dementia has resulted in the development of several communication techniques that are aimed at reducing social isolation through focusing on paralinguistic and nonverbal aspects such as “adaptive interaction” (Ellis & Astell, 2017). Adaptive interaction is based on “intensive interaction” (Nind & Hewett, 2012) and as a central component it uses the process of “mirroring,” where any communication attempts are reflected back to the person initiating them in order to bridge communication difficulties (Henwood & Ellis, 2015).

## Music

Music can facilitate mirroring and has been shown to offer a fundamental, emotion-based connection (Pace, Treloar, & Scott, 2011). Music interventions have also been found to decrease stress hormones (Spintge, 2000), increase relaxation and emotional wellbeing (Brotons & Koger, 2000), provide a sense of safety, and reduce anxiety (van der Steen et al., 2018). Other research has shown that singing groups can enhance a sense of equality for people with dementia and their carers (Unadkat, Camic, & Vella-Burrows, 2017) and that regular musical activities can have long-term cognitive, emotional, and social benefits for people with mild/moderate dementia (Sarkamo et al., 2014).

Group music interventions, specifically, can help prevent social isolation by encouraging social interaction and communication of feelings and ideas (Aldridge, 1996). According to van der Steen and colleagues (2018) group music interventions provide opportunities to make connections with other people through nonverbal musical communication, which may help people cope with their illness and build relationships. Most of the studies looking at group music have, however, focused on measuring biological, psychological, and cognitive symptoms using quantitative analysis rather than looking at relational, communicative, and other positive responses to music (Dowson, McDermott, & Schneider, 2019).

## Video Analysis

Several studies looking at the impact of music on people with dementia and carers have used video analysis (e.g., Engström, Marmstål Hammar, Williams, & Gotell, 2011; Ragneskog, Asplund, Kihlgren, & Norberg, 2001). Many of these studies have used quantitative measures to analyze video data but there have been no studies, found to date, that sought to develop a conceptual understanding of communication using video analysis. According to Konecki (2011) visual grounded theory can be used to generate theories, construct categories, and describe properties, which account for a visual social process. Griffiths (2013) and Griffiths and Smith (2016) used visual grounded theory methodology with people with severe and complex learning disabilities and determined it has the potential to uncover and explain patterns of nonverbal interactions that would not have been revealed previously.

## Aims

This project investigated a live music group based on the Music for Life (Rose, 1993) approach, which uniquely brings together professional musicians, care staff, and people living with dementia through longitudinal interactive music sessions aiming to enhance quality of life. Cooke, Moyle, Shum, Harrison, and Murfield (2010) highlight the importance of individualizing interventions and therefore this study looked at live music facilitated by musicians using improvisation to interact with group participants. The current study built a conceptual understanding around communication that enables us to theorize how people with dementia express themselves nonverbally in response to music and in relation to other group members over time.

Specifically, the research sought to answer the following questions:

1. What is the nature and range of communicative interactions between people with dementia, care staff and musicians within a Music for Life group?
2. How does communication change over the course of the group sessions?

## Research Design and Methods

This study employed a qualitative, longitudinal design influenced by classic grounded theory (CGT) (Glaser & Strauss, 1967) and video analysis as established by Griffiths (2013). According to Griffiths and Smith (2016), the use of visual CGT provides a way of eliciting meaning from people who express themselves primarily using nonverbal means as it allows for careful observation of subtle or nuanced communication. Classic grounded theory was used over other qualitative methods as it supports the development of a theoretical understanding grounded in social experiences (Urquhart, 2013).

### Participants

Participants included eight adults with advanced dementia who lived in the same residential care home (Table 1). Diagnosis was made by National Health Service physicians prior to admittance to the care home. Level of dementia impairment was determined by the clinical dementia rating scale (Morris, 1997). This scale measures impairment from 0 (none) to 3 (severe) across six areas (memory, orientation, judgment/problem solving, community affairs, home and hobbies, and personal care). Additional participants were staff members from the care home (three to five per session) and professional musicians (three each session) trained in the Music for Life intervention.

**Table 1.** Participant Demographics

*Participant*	*Gender*	*Age*	*Type of dementia*	Level of impairment*
1	Female	97	Atypical or mixed	2.5
2	Female	93	Alzheimer’s	2.5
3	Male	92	Mixed– Alzheimer’s and vascular	3.0
4	Male	92	Alzheimer’s	2.5
5	Male	92	Alzheimer’s	2.5
6	Female	82	Mixed- Alzheimer’s and vascular	2.0
7	Male	85	Alzheimer’s	2.5
8	Female	88	Vascular dementia	2.5

*Note*: *Clinical Dementia Rating scale (Morris, 1997).

### Procedure

#### Ethical considerations and consent

An ethics panel at Canterbury Christ Church University approved the study (ID: 18–075). Participants (given pseudonyms) were in the advanced stages of dementia and lacked the capacity to give consent; their legal guardians provided this on their behalves in line with Mental Capacity Act recommendations (MCA; Department of Health, 2005). No family guardian declined to give consent. The British Psychological Society’s Code of Ethics (2009) was followed throughout the research.

### Intervention

The intervention involved 8-hr-long live music sessions adhering to a similar format each week: an opening piece based on a musical theme involving harp, flute, and oboe, followed by the “welcome song,” which was same piece each week led by one of the musicians singing “welcome” to each resident and naming them individually. Improvised group and one-on-one music then followed and continued throughout the session. An ending piece based on the same musical material as the opening piece, completed each session. Musicians and participants played a range of instruments throughout the intervention (Table 2).

**Table 2.** Instruments

Used only by the musicians	Available to the residents, carers, and musicians
Flute	Shakers
Harp	Maracas
Oboe	Drums
	Glockenspiel
	Rain makers
	Tambourines
	Scrapers
	Ocean drum
	Hand chimes
	Chime bars
	Woodblocks
	Claves
	Gato drum
	Tibetan bells
	Cabasa
	Djembe drum
	Small drum

#### Data collection

Video data consisted of one recorded music listening session, used as a “negative case” (Allen, 2017), further described below, followed by 8-hr-long weekly sessions (the intervention). All sessions were recorded using the Fly 360-degree video camera (360fly, Canonsburg, PA, United States) and analyzed by NVivo 11 software (version 11.3.0.773; QSR International, 2016), which allowed slowed-down video analysis (e.g., 0.25 s per frame) to be captured. The Fly 360-degree camera allowed 360-degree footage of the room and a continuous view of all participants, at any point in time, in order to better capture and understand interpersonal interactions.

#### Data analysis

As in Griffiths and Smith’s (2016) study, video was the primary data-gathering tool. Video data consisted of all verbal interactions, nonverbal interactions, observable behaviors and the sequence of interactions. Viewing data from a 360-degree perspective facilitated close observations of simultaneous activity across the group at any given moment as well as easily accessible forward and rewind abilities, thus aiding detailed description throughout.

To begin the analysis, four residents were chosen at random and were then observed for the whole of each session across five of the eight live music sessions (sessions one, three, five, and seven) and the recorded music session. The initial data from these observations helped to guide further theoretical sampling (Urquhart, 2013). A transcription was made of moment-by-moment participant observations: what they were doing, what was happening around them (including the music), as well as what preceded and followed the moment. The transcript was developed by watching each session, slowing down the recording and typing the moment-by-moment observations into a word document. This resulted in a narrative transcript for each of the sessions observed for each participant. Once a narrative transcript was made for each of the initial four participants, the next step involved coding using NVivo-11software. This initial coding involved constant comparison; once the first code was identified, it was compared to the next piece of data in order to see whether they were similar, and could be incorporated together, or whether a new code needed to be named. The codes generated from this initial coding were followed by a process of selective coding where the codes were grouped together into core categories, depending on whether they shared similar properties. Throughout coding, theoretical memos were written to help identify emerging relationships and conceptual categories. The process of coding involved an ongoing shaping and reshaping of the core categories, which contributed to theoretical development as additional data were coded in an iterative cycle. This process resulted in the emergence of a core category—a category to which all the other concepts and categories related. The aim of the analysis was to reach theoretical sufficiency whereby a sufficient depth of understanding is obtained to allow for a plausible theory to develop (Dey, 1999). Once the core category was identified, it was decided that theoretical sufficiency had been reached.

**Figure 1.**
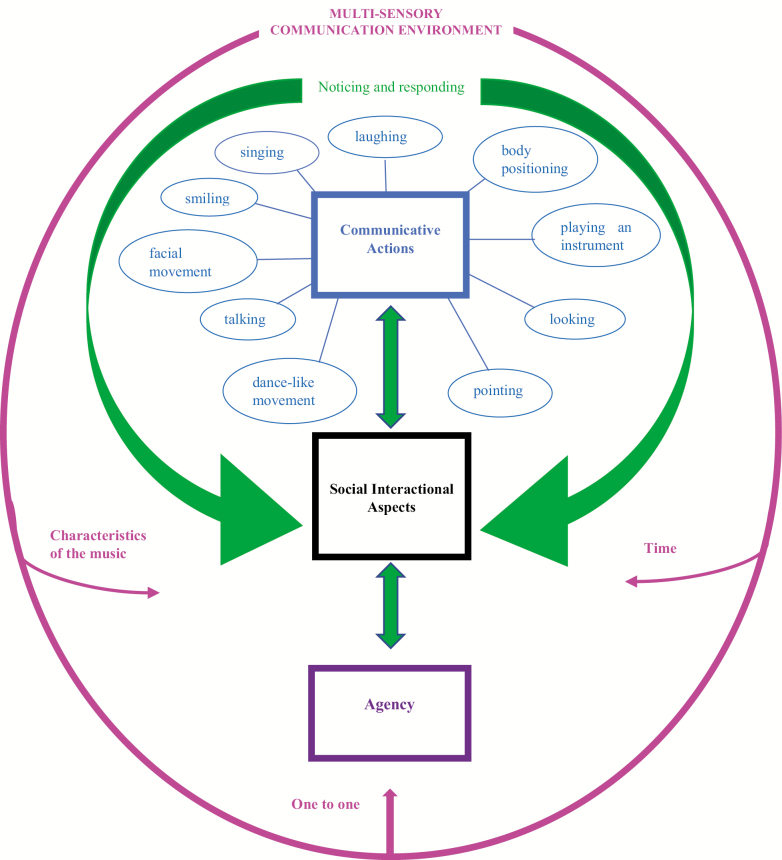
A multisensory communicative environment through music for people with late- stage dementia.

To achieve this, following the initial data analysis additional observation was then completed with the other four residents to test the initial concepts being identified. These latter four residents were not observed for the whole of each of the five sessions. Instead, using theoretical sampling, samples were taken from each session at time periods when the previously observed participants had all shown a noteworthy change (either an increase or decrease, as agreed by AC and PMC) in communicative actions. The coding process was repeated for the additional data, allowing for definition and clarification of categories and further conceptual understanding. Several variations of the emerging theory were developed and adapted as constant comparison took place prior to their consolidation. Once this stage of the analysis was completed, using theoretical sampling, a negative case was chosen, identified where only recorded music was played. The same musicians, staff, and residents also attended this session. The negative case was sampled looking for both similarities and differences across the 10 communicative actions seen in Figure 1. According to Allen (2017), negative case analysis can be used to strengthen qualitative rigor and allow for nuanced analysis and theory development.

#### Quality assurance

Several methods of quality assurance were used in order to monitor and reflect on the researchers’ preconceptions and motivations and the potential impact of this on the analysis. A reflective research diary was completed throughout. The first and second authors examined and discussed all coded data and reviewed corresponding sections of video while all authors discussed and agreed components of the emerging theory. Throughout, Chiovitti and Piran’s (2003) guidelines for grounded theory quality assurance were followed.

## Results

The goal of grounded theory is to identify a core or overarching category that brings together key aspects of the analysis (Griffiths & Smith, 2016). For this study, music making was seen as a way for people to communicate in later stage dementias. The essential overarching aspect of the music-making groups appeared anchored in multiple communicative actions that occurred between musicians and residents, musicians and staff, and residents and staff. These were not linear forms of communicating with a clear beginning and end but were often a network of complex and subtle interactions discovered through close observation. The process that operated to form the core category involving these multiple types of communication is a *multisensory environment* created by the music-making groups. The multisensory nature of the environment provided a unique and supportive communicative atmosphere.

### A Multisensory Communicative Environment Through Music for People with Late-Stage Dementia

The emerging grounded theory is composed of three interacting components: multiple communicative actions, social interactional components, and agency (Figure 1). Multiple communicative actions act as the core category because they included most of the data (Glaser, 1998). The careful, sensitive noticing of and responding to the communicative actions of participants by the musicians set the foundation for the social interactional components of communication (mirroring, turn taking, and humor) and agency (e.g., choosing to sing or be silent) to take place. A unique multisensory environment that was fundamental to the group supported the development and maintenance of the three components.

### Structure of the Grounded Theory

#### Multiple communicative actions

The data consisted of all observable behavior of the participants across the music sessions. The data were coded and categorized into 10 communicative actions: laughing, body positioning, playing an instrument, looking, pointing, dance-like movement, talking, facial movement, smiling, and singing. A description of the communicative actions and sample codes are shown in Table 3. Each action involved a level of physicality in that they involved movement of all or certain parts of the body and contained either nonverbal or verbal components. They formed the core category of communicative actions because, as theorized by Argyle (1988), any verbal or nonverbal behavior that has the potential to influence another individual, intentionally or not, can be seen as communicative. Within the groups, it seemed that each of these actions had the ability to influence the behavior of the musicians or staff and therefore moved from being solely a physical action to a communicative action.

**Table 3.** Multiple Communicative Actions and Sample Codes

Subcategories	Description	Codes
Talking	Observations of the residents talking characteristics of the music	Talking at any time
Smiling	Residents smiling, who they were smiling at and what was happening around them at the time as described above	Smiling when leading music. In response to 1:1, the welcome song, own name being sung, a smile from another person
Singing	Singing to another person and singing with other people. This was considered communicative when it involved other people, eye contact and the potential to influence other people’s behavior).	Singing to self. Singing in response to own name being sung. Singing along to welcome song, singing more words each week, singing louder each week
Pointing	Residents pointing, what they were pointing at, whether they were *also* looking at another person to direct their attention and what was happening around them at the time.	Pointing to a person talking, an instrument, other people in the room.
Playing an instrument	A resident playing an instrument by themselves, during music, with support from someone else.	Playing an instrument in response to gentle music, to lively music, music with an increased tempo.
Looking	Observations of where the residents were looking towards and what was happening around them.	Looking at others talking Looking at other people during recorded music or live music
Laughing	The residents laughing at what was happening around them at the time.	Laughing response to: a carer; during 1:1 interaction; in response to an action from someone else.
Facial movement	Observations of parts of the residents’ face moving and what was happening around them at the time.	Raised eye brows in response to eye contact and smiling from carer. Facial movement whilst playing an instrument and listening to music.
Dance-like movement	Residents moving in time to the music.	Legs, hands, or arms moving in time to the music as though doing dance steps to up tempo and lively music.
Body positioning	How the resident was seated and any nonspecific body movement during the group and what was happening around them at the time.	Mouth covered while singing and becoming uncovered over time during welcome song. Leaning back and forth during music. Fidgeting during recorded music (lots of little movement and changes to positioning).

#### Noticing and responding

If a communicative action by a resident was noticed it could be responded to by a musician, other resident, or staff member. A communicative action being responded to often led to occurrences of other communicative actions, for example, laughter from a resident leading to laughter from a carer and eye contact. There were times when the communicative actions were not noticed, for example, if the musicians were focusing on a different resident; it was observed that at these times the residents tended to cease the communication attempt or turn their attention to objects around them such as their instrument or their clothes. For example, an interaction with Anne in session seven was observed as follows:

*Staff member turned away, [Anne] sitting stiller, looking at baton, looking towards staff member, smiling at her [staff member still turned away] looking down.*

Noticing and responding to both verbal and nonverbal methods of communication, were essential because they allowed for the second component of the theory to develop. Yet it is also relevant to point out there are some situations where a response is not expected or required and its absence does not necessarily reduce further communication.

### Social Interactional Components

#### Mirroring

The noticing and responding to the communicative actions allowed for mirroring and turn taking to occur with the musicians modeling these processes within their collaborative music making (Table 4). The musicians used mirroring as part of their practice be it through body positioning, vocalizing, or through the use of their instruments.

**Table 4.** Social Interactional Components and Sample Codes

Aspect	Description	Codes
Mirroring	A communicative action reflected back to the initiating person The mirroring might be done by a resident, carer, or musician.	Mirroring the type of instrument, how it is being held, the sound being created and the character or the number of beats. Mirroring facial movement (e.g., smiling or eye contact), singing, body positioning, or movement (e.g., leaning forward when someone else does).
Turn taking	A back and forth interaction between the residents and the musicians or staff.	Turn taking of spoken words, of singing, while playing instruments and during dance-like movement.
Humor	The use of what appeared to be playful communicative actions that led to other people laughing.	Using gesture to make people laugh. Playing an instrument in an increasingly faster or complicated beat that was trying to be mirrored by the musician and smiling whilst doing so. Laughing together following a turn taking exchange with the instruments.

Jim, session five: *given wood block by person sat next to him, he starts to tap it and musician starts to play the drum mirroring the rhythm. Jane, session seven: musician holding an instrument with Jane and she starts making mouth movements, possibly vocalising, musician mirrors facial expression and mouth movements back to Jane with vocalisations matching her attempts to make sounds*

#### Turn taking

Turn taking in this study related to a nonverbal or verbal back and forth exchange between two people. Mirroring entered into this process as a way of initiating and prolonging an interaction. Turn taking was enabled by the processes of noticing and responding to the multiple types of communication. The musicians would often use their instruments to start a turn taking exchange in response to the resident playing an instrument.

Tom, Session seven: *Tom hits xylophone - each bar in order. Musician responds by mirroring notes and rhythm of this with oboe, Tom then hits the bars again and the musician responds, turn taking exchange continues. Harry, session seven: music stops, tapping fingers in rhythm on drum in silence whilst smiling and sticks tongue out at musician. Takes it in turn with musician to imitate each other’s drumming. Smiling and eye contact with musician. Lifts up hands when had enough as in ‘I’m beaten’ and smiles – everyone laughs.*

Both mirroring and turn taking processes require a negotiation by both communication partners and a careful noticing, inviting, or stepping back in response to subtle cues.

#### Humor

Humor was seen as a social interaction because it required more than one individual and relied upon another person noticing and responding to its use. It involved a complex cognitive process: gaining other people’s attention, deciding what to say or do, and carrying this out with the intention of making others laugh. This humor was evident in both verbal and nonverbal behaviors and often had a playful aspect to it. Harry used humor on several occasions, utilizing both verbal and nonverbal means, a particular incident was during the welcome song of session five and the following occurred:

*Staff member sang his name – Harry said ‘who me’ whilst pointing at himself, people laughed, he smiled and laughed and jokily looked inside his jacket for someone else and then looks at musician.*

#### Agency

According to Boyle (2014), agency is “the ability to initiate social action or at least inﬂuence own personal circumstances” and can be indicated by both behavior and emotion. Agency (Table 5) was evident throughout the group through the residents having the opportunity to choose how to participate but also through the opportunity to influence the music being created. The musicians were essential to the development of agency (see negative case) through their noticing and responding to the communicative actions of the residents, providing opportunity for social interaction through mirroring, and turn taking as well as inviting the residents to participate in and influence the music being made. As a result, the residents could choose how they interacted with the group and the individuals within it. Participating in the group did not rely on a person playing an instrument and several of the residents chose to interact in other ways as shown by the range of communicative actions observed. For example, Mark spent time with his eyes shut, however, small changes to his facial musculature and his eyes flitting between being open and closed indicated he was not asleep, as shown in session one:

**Table 5.** Descriptions of Agency and Sample Codes

Description	Codes
The ability of the residents to influence social action or personal circumstances through the use of behavior. The choice of how to participate. The choice of influencing the music.	Being able to sit in the group with eyes shut. Choosing to sing along or sit in silence, to take an instrument, and/or to hand it back. Declining an instrument. Putting down an instrument.

*Eyes shut whilst instruments are handed out. Lively music – brisk with oboe – eyes open and mouth less slack. Watching musician across the room introducing an instrument to a resident. Percussion comes in. Eyes shut but mouth tight.*

Some of the residents showed agency via how they wanted to interact by choosing to hand back or put down an instrument, for example:

Harry, session three: *handed an instrument by musician, they are talking about it and he is turning it over then he hands it back to musician. She returns it to him and points to show another musician demonstrating it. He hands it back to her.*

At other times, residents were able to show their agency in the groups by being given the opportunity to direct the music by the musicians (e.g., responding to their communicative actions or inviting them to create a sound by demonstrating how to play an instrument, verbal encouragement or mirroring). This was shown by the examples of turn taking and also in the following observations:

Tom, session five: *Tom starts hitting a wooden block with a mallet, the musician joins in gently tapping the drum and mirroring the rhythm Tom is playing. The flautist joins in following this rhythm. Other residents start playing instruments in time or tapping in time. Tom stops and the music stops. There is talking and then Tom is invited to start playing again, he does so, and the musicians follow his rhythm again.*

### Multisensory Environment

An overarching multisensory environment involving communicative actions, social interaction and agency, was a fundamental aspect of the intervention. It is multisensory in the sense that it contains visual, auditory, physical, and tactile components. The instruments could be seen, heard and touched; everyone was seated in a way that allowed them to see, hear and potentially physically interact each other (e.g., see facial expressions, body movements, see and hear laughter, and spoken words). The multisensory environment reinforced that the group accepted and responded to a variety of different means of communication that relied upon the use of different senses. It also allowed social interactions to occur, as well as the opportunity for agency, using a variety of communicative actions. It gave the residents the opportunity to make use of and interact in ways that appealed to a variety of senses.

Within this multisensory environment, there were three factors that influenced the three main components of the theory. These emerged as theoretical memos during the data analysis following observations that the communicative actions for all the participants tended to change in response to the same factors: time, one-to-one interaction and the characteristics of the music.

#### Time

Time related to experiencing the multisensory nature of the group on a weekly basis, particularly those aspects of the group that were repeated or similar every week: the music at the beginning as the group when settling in, the welcome song and the music at the end. The familiar melodies used may have orientated the residents to the purpose of the group or acted as a reminder of what happens and how they can participate leading to a change in observed communicative actions over time. This change indicated that some of the residents had remembered parts of the routine aspects of the group as seen by singing along to the words or using more exaggerated movements in each successive session. For example, Jane during the welcome songs in session one where she *sat with eyes shut, mouth open and sitting back* and then in session seven where she was *sitting back, eyes wide open, mouthing along to the chorus whilst looking at the musician.* Another participant, Harry, sat during the first session with his hand over his mouth watching the musicians. As each session passed, he uncovered his mouth a little more until by the seventh session he was singing along with his hands in his lap.

#### One-to-one interaction

This aspect related to the residents having time where they were receiving direct attention from another individual (e.g., a carer or a musician). The one-to-one interactions in the group were multisensory in nature involving physical closeness, eye contact, clearly visible facial expression, playing of instruments, touch, and spoken words. These interactions seemed to lead to changes in communicative actions for all of the residents.

Anne, session seven: *music has stopped and talking, Anne has been looking down at instruments for a while, torso still, foot tapping. Musician looking at and moving to be in front of Anne. Anne looks up. Given a drum, Anne says ‘yes’ to musician asking, ‘shall we play together?’. Anne starts hitting the drum quickly and loudly, smiling, muscles tense. ‘Too loud?’ she says frowning, ‘I don’t know?’; musician plays the djembe drum following her rhythm – she sits back a bit and keeps hitting it, now smiling. Harp joins in, she starts playing quicker. Musician sings her name next to her, she suddenly looks up and smiles at her.*

There were many instances of these changes but there were also some occasions where a decrease in communicative actions was observed when the person giving the attention turned away:

Jane, session five: *Musician has turned to face Jane, sat next to her, sings her name and holds her hand. She turns to face musician, makes eye contact and smiles. Musician turns away and Jane shuts her eyes and smile fades.*

#### Characteristics of the music

These are related to the tempo, character, and type of instrument used to create the music at a set moment. Tempo refers to the music’s speed whereas character refers to its nature, for example, whether the music was gentle, brisk, smooth flowing, or lively or a combination of these. The type of instrument (Table 2) refers specifically to which instrument was playing the predominant line or which instrument seemed to lead to changes in communicative actions when it joined in the music. A larger drum, known as a djembe drum, in particular seemed to lead to a change in communicative action for most of the residents.

The character of the music making was multisensory in nature; instruments could be felt and handled and the music could be heard but also seen through body movements and facial expressions of other people in the room. When the tempo, character, and type of instrument interacted in certain ways, it seemed to produce more changes in communicative action than at other times, specifically when the tempo was faster and the character was lively at the same time when the djembe drum being played.

Mark, session five: *eyes drooping, minimal music in the room. Passing instrument back to member of staff. Music gentle. Watching flautist. Increase in tempo and music becomes lively – does not take offered instrument but smiles and laughs. Djembe drum next to him starts being hit. Some nodding, starts to drum on legs in time as music picks up speed. Given shaker, starts tapping it on head in time. Jane, session five: Jane is sat with her eyes closed, music is building through different instruments joining in, tempo increases, lively and brisk, flute playing predominant line, djembe drum joins along with other percussion in the room (other resident playing a tambourine), Jane opens her eyes and looks at musicians, keeps her eyes open, looks at resident playing tambourine.*

### A Negative Case

A session involving the same participants but listening to recorded music was analyzed following the method described above. This played an important part in the theory’s development because the observations during this session did not seem to fit the developing grounded theory. In relation to the multiple communicative actions, it was notable that there were fewer observations for most of the subcategories except for “talking” which increased. For example, one resident (Anne) spent most of the session talking aloud, she did not appear to be talking to anyone in particular as indicated by her lack of eye contact to anybody in the room. When another person responded to her verbally she continued to talk aloud without looking at them. In addition, there were fewer observations of mirroring, turn taking or humor. In contrast to the live music, some residents tried to stand up as though to leave the room, which may have been a way of showing agency by choosing to leave or demonstrating disinterest. With these observations in mind, this study proposes that the live music carried a unique quality by involving a multisensory communication environment.

## Discussion

This study sought to develop a conceptual understanding of communication within a group intervention, Music for Life, for people with advanced dementias in residential care. In doing so, it attempted to understand the ways people with dementias communicate longitudinally over an 8-week music-based activity. Furthermore, it sought to draw out relationships that may exist between the way people communicated and the actions and processes of the music, musicians, and carers.

The communicative actions observed included both verbal and nonverbal. Verbal communication can be compromised for people with dementia therefore reducing the opportunities for making connections with other people, building relationships, sharing experiences, and belonging to a group. This is particularly the case if the environment they live in places highest value in verbal communication. According to Ridder (2003), it is essential that both verbal and nonverbal communication are attended and responded to for people with dementia allowing for social interactions and therefore relationships to develop. This study supports this view and indicated that with careful noticing and responding to both verbal and nonverbal communicative actions a social interaction can occur through the use of mirroring and turn taking.

Caldwell (2008) suggests that mirroring is “a way of capturing attention, a door to enter the inner world of our partners” (p. 72). Previous research has shown that nonverbal communication techniques have been developed that use mirroring as their core foundation; intensive interaction (Nind & Hewett, 2012) and the subsequent version for people with dementia, adaptive interaction (Ellis & Astell, 2017). Both have been shown to improve quality of life, increase nonverbal communication repertoire and allow for connections to be built between people with communication impairment (Ellis & Astell, 2017). The musicians’ use of mirroring and turn taking during the group may offer the same benefits and support Wigram’s (2012) research that music can help to develop a nonverbal conversation between two people.

The communicative actions and the subsequent social interactional components align with previous research indicating the importance of relationship-centered care. In doing so, interactions can be developed in order to reduce social isolation (Nolan et al., 2004). The theory aligns closely with Nolan et al.’s (2004) “senses framework” that suggests that all those involved in caring, as well as people with dementia, need to experience relationships that promote a sense of security, belonging, continuity, purpose, achievement, and significance.

The multisensory nature of the intervention supports previous research that indicated multisensory environments can improve communication between staff and residents (van Weert, van Dulmen, Spreeuwenberg, Ribbe, & Bensing, 2005). Furthermore, several of the residents showed that they had remembered certain routine parts of the group indicating that a musical multisensory environment may be able to enhance new learning in advanced dementia.

This study also offers insight into the changes in expressions of agency. According to Boyle (2014), people with dementia have been historically assumed to have little or no agency as a result of agency theory being heavily influenced by verbal abilities. Boyle (2014) argues however that agency can be demonstrated through the expression of emotion and nonverbal behavior. This study supports this argument in that residents were given several opportunities to make choices: a choice to participate from the onset, of how to participate, whether (and how) to influence the musicians’ actions, and about the music being created (Camic et al., 2018).

## Implications

### Methodology

According to Griffiths and Smith (2016), the analysis of microdata from detailed descriptors of verbal and nonverbal interactions using video recorded data allows for the theorizing of behavior that may usually be unremarkable, small, or unseen. The use of video analysis in this study allowed careful observation of subtle communicative actions and the 360-degree camera provided in the moment observation of multiple, simultaneous interactions, permitting comparisons between participants. These nuanced interactions and behaviors would likely have been missed if a camera with a more limited field of view had been used or only field observations had been made. The ability to slow the recording, pause, go back, and review specific moments allowed for a detailed analysis of behavioral and interactional aspects.

### Music-Based Interventions

The majority of the interactions occurred between the musicians or carers and the residents with dementia. It may be that the residents could be encouraged to direct their instrument playing towards each other to increase the group’s interaction as a whole. It is interesting that the character of the music seemed important to the communicative actions observed. Specifically, the tempo, nature, and type of instrument used can have an impact on the way that people communicate. In this study, lively music of a faster tempo led to changes in communicative actions particularly when the djembe drum was used. The fact that all of the music being played was improvised, with musicians responding moment by moment was of significance; playing set pieces of music would not have allowed the residents to influence the music making to the same degree.

### Dementia Care

Staff and relatives could be encouraged to notice and respond to nonverbal communication that may be very subtle or unremarkable in day-to-day life. By doing so they may be able to develop social interactions and therefore strengthen relationships with people with dementia. Live music that responds to individual communicative action within a multisensory environment can be a way of responding to and building social interactions for people who may find verbal expression difficult. Taken with other studies involving music and singing in the early to middle stages of dementia, using live music in residential care may be able to reduce isolation, increase enjoyment for staff and residents, and encourage types of communication that could increase positive interactions in residential care environments. This study also indicated that one-to-one interactions within a group environment led to positive changes in communicative actions and social interactions between both staff and residents and residents and musicians.

### Limitations

The nature of the methodology and the detail of observation performed was essential for observing the nuanced behaviors and the subtle interactions surrounding them. Although time consuming, according to Griffiths and Smith (2016), this is not necessarily a limitation but instead a caution about the methodology. The timing of the negative case (the recorded music session), being prior to the live music sessions, may have influenced the differences in observations that were noted for that session. During that first session, the participants would have been less familiar with the musicians and the format.

A larger-scale study may be able to gather data from similar groups in different residential care settings involving people from different socioeconomic or ethnic backgrounds. Although grounded theory assumes that the theoretical understanding applies to the context it is derived from, the current sample meant that it was not possible to feel certain that this intervention would result in a similar analysis in a different setting with other participants. There was also a possibility of the influence of the effect of the researchers’ preconceptions and assumptions, although these were discussed and reflected on in team meetings and through a research diary.

### Future Research

Further research should consider including the carer staff and musicians’ experiences of being in the group and how the group might further support the senses framework suggested by Nolan and colleagues (2004). This framework suggests that all parties involved in caring need to experience relationships that promote a sense of security, belonging, continuity, purpose, achievement, and significance. It seems likely that the intervention described in this study could encourage the development of relationships between residents and care staff and therefore help to promote relationship-centered care outside of the group.

## Conclusion

This study lent support to the notion that people with advanced dementias were active communicators in a variety of verbal and nonverbal ways within a multisensory live music-based communicative environment. When these communicative actions were noticed and responded to through the use of careful observation by the musicians, it led to a positive social interaction taking place through the use of mirroring, turn taking, and humor. In this way, people with dementia were given the opportunity to make a choice as to how to participate and to influence the creation of the music and the “musical conversation”; by doing so people with dementia experienced agency. The developing grounded theory of a multisensory communicative environment through music for people with late-stage dementia supports relational theories of dementia care (e.g., Nolan et al., 2004).
